# PWAS Hub for exploring gene-based associations of common complex diseases

**DOI:** 10.1101/gr.278916.123

**Published:** 2024-10

**Authors:** Guy Kelman, Roei Zucker, Nadav Brandes, Michal Linial

**Affiliations:** 1 The Jerusalem Center for Personalized Computational Medicine, Faculty of Medicine, The Hebrew University of Jerusalem, Jerusalem 9112102, Israel;; 2 The Rachel and Selim Benin School of Computer Science and Engineering, The Hebrew University of Jerusalem, Jerusalem 91904, Israel;; 3 Division of Rheumatology, Department of Medicine, University of California San Francisco, San Francisco, California 94143, USA;; 4 Department of Biological Chemistry, Institute of Life Sciences, The Hebrew University of Jerusalem, Jerusalem 91904, Israel

## Abstract

PWAS (proteome-wide association study) is an innovative genetic association approach that complements widely used methods like GWAS (genome-wide association study). The PWAS approach involves consecutive phases. Initially, machine learning modeling and probabilistic considerations quantify the impact of genetic variants on protein-coding genes’ biochemical functions. Secondly, for each individual, aggregating the variants per gene determines a gene-damaging score. Finally, standard statistical tests are activated in the case-control setting to yield statistically significant genes per phenotype. The PWAS Hub offers a user-friendly interface for an in-depth exploration of gene–disease associations from the UK Biobank (UKB). Results from PWAS cover 99 common diseases and conditions, each with over 10,000 diagnosed individuals per phenotype. Users can explore genes associated with these diseases, with separate analyses conducted for males and females. For each phenotype, the analyses account for sex-based genetic effects, inheritance modes (dominant and recessive), and the pleiotropic nature of associated genes. The PWAS Hub showcases its usefulness for asthma by navigating through proteomic-genetic analyses. Inspecting PWAS asthma-listed genes (a total of 27) provide insights into the underlying cellular and molecular mechanisms. Comparison of PWAS-statistically significant genes for common diseases to the Open Targets benchmark shows partial but significant overlap in gene associations for most phenotypes. Graphical tools facilitate comparing genetic effects between PWAS and coding GWAS results, aiding in understanding the sex-specific genetic impact on common diseases. This adaptable platform is attractive to clinicians, researchers, and individuals interested in delving into gene–disease associations and sex-specific genetic effects.

Public databases have compiled thousands of reports from genome-wide association studies (GWAS) (MacArthur et al. 2017). Despite their abundance, most GWAS have not significantly contributed to our understanding of disease mechanisms (Visscher et al. 2012; Brandes et al. 2022). Mapping variants to their respective credible genes is challenging in traditional GWAS. This may lead to ambiguity and often unjustified variant-to-gene associations (Schaid et al. 2018). Notably, a large proportion of GWAS-associated variants (e.g., from the GWAS catalog or the GWAS central) may occur in intergenic regions or in regions that are remote from any gene (coding or noncoding). While variants in such regions may affect chromatin structure and gene expression regulation, sparse functional annotations make it difficult to gain meaningful insights into disease etiology (Mountjoy et al. 2021).

To address these gaps in contemporary genetic studies, we developed PWAS (proteome-wide association study), a method that conducts genetic association (GA) studies at the gene level (Brandes et al. 2020). PWAS aims to infer causal associations from variations within protein-coding genes. It utilizes a scoring method based on machine learning (ML) and probabilistic modeling to assess the functional effects of genetic variants and their impact on protein-coding genes (Brandes et al. 2019). The development of PWAS involved utilizing genotyping data from the UK Biobank (UKB) (Zhao et al. 2020). The input for PWAS includes all tagged and imputed SNPs within the coding regions of protein-coding genes. The underlying idea is that by aggregating weak effects across different variants, PWAS can detect genetic signals at the gene level. Using a gene-damage probability score per individual, robust and confident associations are identified by applying standard statistical methods. Specifically, linear regression models test associations for continuous phenotypes (e.g., BMI, height), while logistic regression models are used for binary phenotypes (e.g., schizophrenia, asthma). Notably, PWAS accommodates nonlinear genetic effects by considering dominant or recessive inheritance models for each associated gene (Brandes et al. 2020). PWAS complements GWAS and other gene-centric approaches like SKAT (Wu et al. 2011), providing interpretable results by focusing on associations reliant on functional alterations.

Traditionally, sex differences in complex human traits were limited to physical and behavioral differences. They were attributed mainly to the influence of sex chromosomes and gene interactions with sex hormones. These differences translate to anthropometric measurements (e.g., height, weight, adipose in body parts), but to a lesser extent to human physiology and the mechanisms of complex traits (Gilks et al. 2014). However, the exploration of sex-dependent genetics in human traits has become feasible in recent years due to the increased scale of resources with rich genomic and clinical data (Bernabeu et al. 2021).

The PWAS Hub leverages the results of PWAS for most of the abundant complex diseases and conditions represented in UKB (e.g., asthma, hypertension, type 2 diabetes). It encompasses the human coding genome, covering over 18,000 protein-coding genes, with each gene tested according to dominant, recessive, and combined inheritance models. PWAS Hub provides cross-references to relevant resources for genomics, proteomics, and phenotype-related information. It includes a user-friendly website with browsing capabilities for genes and medical outcomes. We conduct separate analyses for females, males, and both sexes, allowing interactive navigation across sexes and by the specific model of inheritance.

## Results

### Gene-based associations for common diseases

The PWAS Hub is a portal presenting an in-depth statistical analysis of findings according to the PWAS framework (Brandes et al. 2020). Further details on the underlying methodology can be found in the original publication (Brandes et al. 2020). The PWAS Hub covers binary complex diseases (e.g., primary hypertension, International Classification of Diseases, Tenth Revision [ICD-10]: I10) and phenotypes (e.g., obesity, ICD-10: E66), with at least 10,000 affected individuals (cases) and a large number of controls ranging from 138k to 264k (Table 1).

**Table 1.** Samples of the Summary page from the PWAS Hub (phenotypes are partitioned by sex)

ICD-10	Disease/phenotype	Sex	# of cases	# of controls	# of PWAS sig. genes	Prevalence (%)	Ratio F:M
E03	Other hypothyroidism	M	2566	123,075	9	2.04	
		F	11,121	138,062	63	7.45	
		Both	13,687	261,137	77	4.98	3.65
I10	Essential (primary) hypertension	M	40,358	85,283	2	32.12	
		F	33,732	115,451	22	22.61	
		Both	74,090	200,734	70	26.96	0.70
R31	Unspecified hematuria	M	8075	117,566	8	6.43	
		F	5694	143,489	26	3.82	
		Both	13,769	261,055	37	5.01	0.59
I25	Chronic ischemic heart disease	M	17,853	107,788	19	14.21	
		F	6963	142,220	0	4.67	
		Both	24,816	250,008	15	9.03	0.33
E780	Pure hypercholesterolemia	M	19,586	106,055	16	15.59	
		F	12,490	136,693	6	8.37	
		Both	32,076	242,748	27	11.67	0.54
E11	Type 2 diabetes mellitus	M	11,098	114,543	12	8.83	
		F	6791	142,392	3	4.55	
		Both	17,889	256,935	38	6.51	0.52

The original PWAS publication reported on 49 representative phenotypes (Brandes et al. 2020), and many of them were based on continuous measurements (e.g., BMI, intraocular pressure, mean corpuscular volume) without a threshold on the minimal number of cases. Additionally, the current PWAS Hub utilizes an updated version of the UKB data (from 2023) with an average of ∼20% increase in the number of cases per phenotype.

The PWAS Hub reports on 86 diseases and binary phenotypes. Each phenotype was tested independently following a procedure of grouping the population into females, males, or both. Table 1 lists a sample of phenotypes partitioned into males (M), females (F), and both sexes, along with the number of associated significant genes (54% females). The number of “both” (274,824) only covers individuals of European origin, where family-related individuals were filtered out. Based on these numbers, we estimate that the PWAS Hub covers common phenotypes and diseases with >3% prevalence. Note that for some phenotypes, the prevalence of males and females is substantially different. For example, while hypothyroidism shows a 3.6-fold higher occurrence in females versus males (Table 1) in chronic ischemic heart disease the ratio of M/F is 3.0. We observed that for most diseases, the number of PWAS-significant genes without the sex partition (i.e., both) is larger relative to the genes discovered according to sex. For example, there were 38 significant genes for ICD-10 E11 (type 2 diabetes mellitus) but only 3 and 12 for the female and male groups, respectively. An exception is ICD-10 I25 (chronic ischemic heart disease), where only 15 genes were associated with the entire cohort, but 19 genes were reported for males and none for females.

Table 1 provides a comprehensive view of population prevalence and the ratio of females to males per a sample of diseases. An extended table can be viewed on the PWAS Hub portal's “Summary page.” Details on the ICD-10 vocabulary are provided via a direct link at the top panel of the “Summary page.” The summary view highlights the genetic effects that resulted in different numbers of PWAS-associated genes for females and males. The full table can be found at https://pwas.huji.ac.il/service.

### Layout of PWAS Hub

The PWAS Hub is accessible at https://pwas.huji.ac.il. We provide an entry point to PWAS Hub through a welcome page that summarizes the actions that can be performed. The user is thus presented with multiple actions in the form of a “What can I do here?” panel. The panel provides a brief overview of the portal utility. The user can select a gene of interest (GOI) as a starting point while seeking associations with any of the diseases listed in the Summary table (as in Table 1). Alternatively, the user may choose a disease of interest (DOI) to estimate its gene-based associations and gain insights toward causality, disease mechanisms, the most informative heritability model, and the sex-dependency of genetic effects.

**Figure 1.**
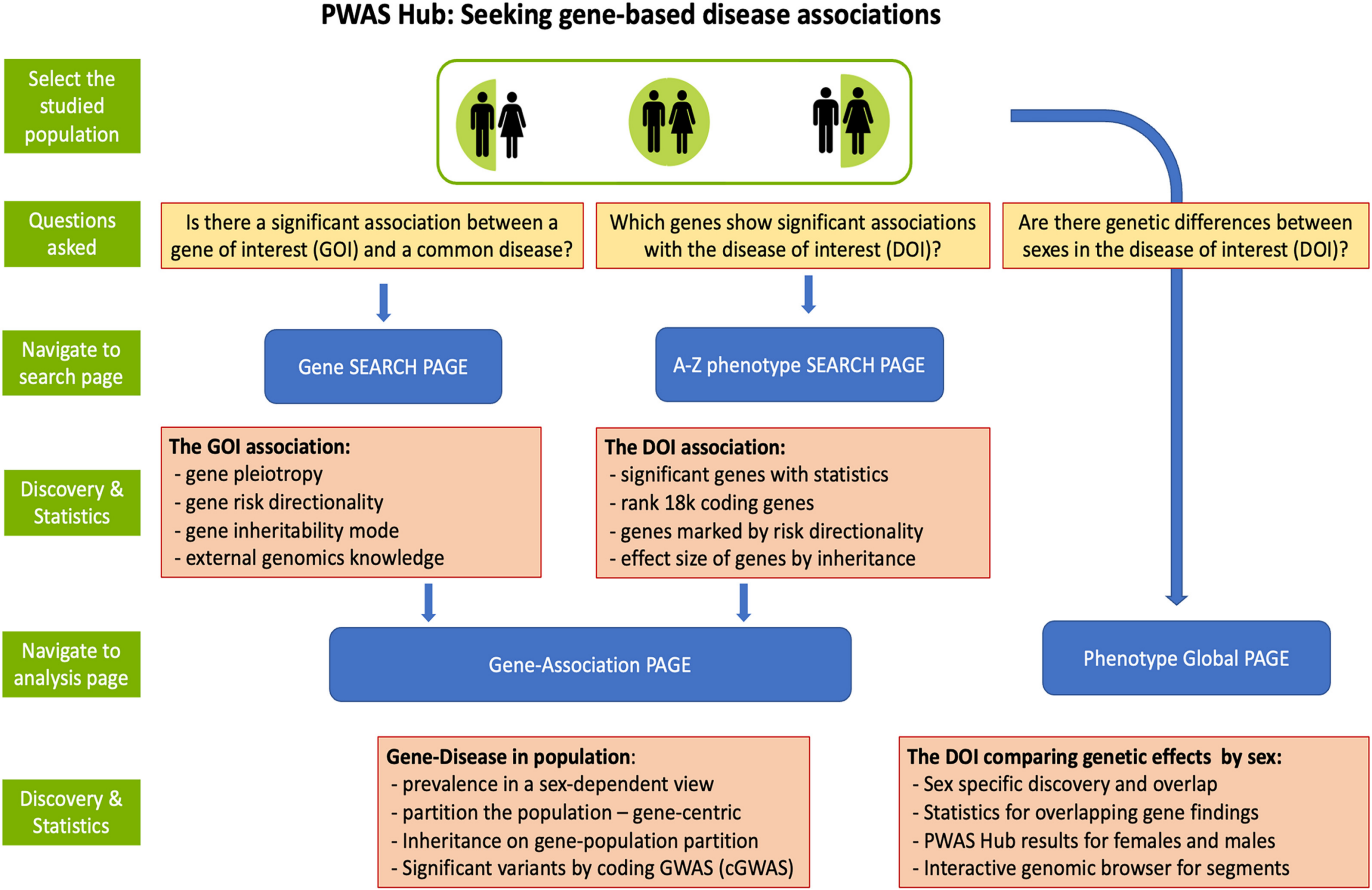
PWAS Hub layout. The primary option for activating the PWAS HUB portal is by selecting cohorts by sex. Then, three questions can be asked as described. The navigation to the “Search page” and “Analysis page” is shown. The main discoveries and supporting statistics are listed in the detailed orange frames associated with each of the major pages of PWAS Hub.

Figure 1 provides an operational workflow for the PWAS Hub. The portal highlights the centrality of sex-dependent genetics by beginning with the desired cohort of females, males, or both. At any stage, the user can compare the sex-based results by switching between the selected cohorts using the green male/female icon. This button appears on any page that offers sex-based analysis. We introduce three main questions that the user may choose to address using the PWAS Hub: (1) focusing on a gene, what conditions is it associated with? (2) focusing on a phenotype, what are the genes that promote or lower its risk? and (3) given a phenotype, what would be the genetic differences across sexes and consequently their sex-oriented risk factors?

### Navigating the asthma showcase in the PWAS Hub

**Figure 2.**
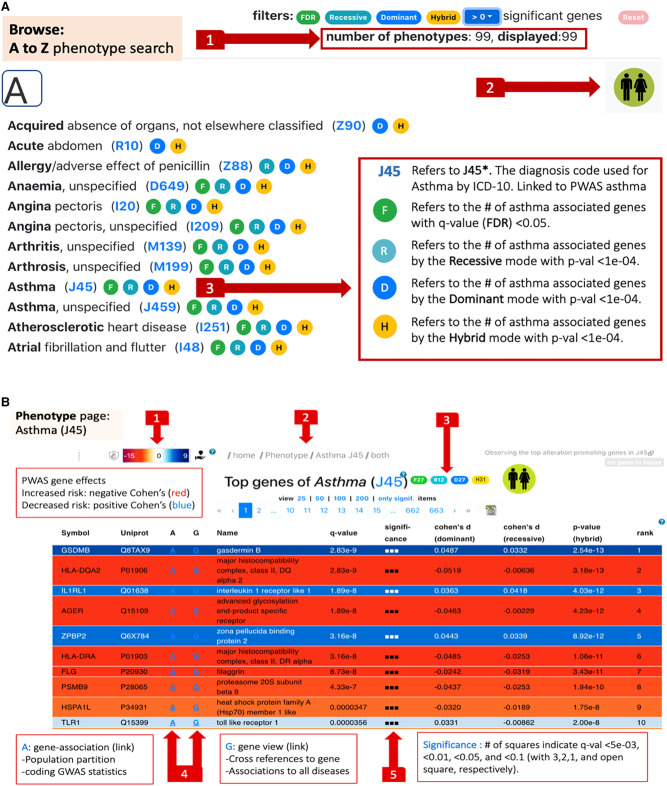
Search page and phenotype page. (*A*) Browsing the “Phenotypes A to Z” search page. The user can filter out phenotypes by selecting a threshold for the number of significant results. Once filtered, (**1**) reports the subset size. The user selects whether the browsing and downstream analyses are restricted to females, males, or both. In this example, a cohort with both sexes was selected (**2**). Each phenotype is indicated by its ICD-10 code (clickable links to Phenotype pages). Results that meet the preselected criteria are marked by the symbols abbreviated F, R, D, and H (**3**). (*B*) The phenotype page for J45 (asthma). The effect sizes of the genes are color-coded from red (negative Cohen's *d* values = increased risk) to blue (positive Cohen's *d* values = decreased risk) (**1**). The intensity of the colors marks the statistical significance (*Q*-value). Breadcrumb navigation links are available on all pages (**2**). Summary statistics of the number of results according to the F, D, R, and H definitions, with an active link to the selected cohort. The link that marks J45 points the user to the “Phenotype global page” (**3**). Navigation to either the “Gene page” (G) or the “Gene-Association page” (A) (**4**). The significance of the listed genes is marked by a predetermined *Q*-value symbolic scale (**5**).

In this section, we illustrate the usability of the PWAS Hub as a navigation and discovery tool. We start by browsing the database using the “Phenotypes A to Z” search page (Fig. 2A, item 1). The layout of the resulting search for the letter A in the alphabetical index is shown (Fig. 2A). The results of the “Phenotype A to Z” search allow an overview of all PWAS gene associations, where for each phenotype, the number of significant genes is reported by colored symbols (acronym F for FDR statistics). The symbols report results by considering inheritance models (acronyms D, R, and H indicate dominant, recessive, and hybrid inherent modes, respectively; see section “Effect size of disease associations”). Statistical thresholds of the number of results according to the F, D, R, and H definitions are presented for each phenotype (Fig. 2A, item 3). Here, we showcase the flow for asthma (ICD-10: J45).

The cohort selection (females, males, or both) is “sticky” in the sense that it carries over to all pages. At any time, the user may switch to a different cohort by using the sex icon button (top right; Fig. 2A, item 2).

The significant gene list for asthma (J45) is composed of 27 genes. Figure 2B gives the resulting top 10 genes identified as significant. We also report on gene candidates with a relaxed threshold (*Q*-value <0.1; 42 genes). The rows are colored to easily assess the genes that foster the greatest chance of increasing or lowering the risk. The range of colors marks Cohen's *d* values with a red color to indicate an increased risk, whereas the blue color represents positive Cohen's *d* values for a decreased risk (Fig. 2B, item 1). The effect sizes of the genes are coded by the color intensity according to the statistical significance (*Q*-value). The table of the associated genes can be sorted by Cohen's *d* value for ranking protective (i.e., reduced risk, colored blue, 11 of 27) and increased risk (16 of 27, colored red) significant genes.

Using the paging tool, the user may also choose to visualize either a significant set only (27 associated genes for asthma) or page through lists of predetermined size with 25, 50, 100, or 200 associated genes. Importantly, statistical analysis for each phenotype is available for all 18.1k analyzed genes. Each row in the table contains navigation links to the gene page or the gene-association page (Fig. 2B, item 4).

To allow a flexible inspection of the gene association with asthma, the gene association table (Fig. 2B) is downloadable and sortable. This can be ranked by information concerning the gene effect size (determined by Cohen's *d* values), where the negative and positive values are associated with an increased or decreased risk, respectively. In addition, significant genes can be sorted by the inheritance signal of choice (dominant, recessive, or hybrid).

For additional support and to enhance the utility of the portal, we refer the user to “help and examples.” To learn more about its applicability, we invite the reader to browse through the tutorial (pwas.huji.ac.il/#tutorial-head), and FAQ (pwas.huji.ac.il/FAQ) sections of the portal.

### Functional interpretation of PWAS-significant genes for asthma

We questioned whether the PWAS-associated genes for asthma can provide biological insights in view of the fact that the PWAS methodology is restricted to the coding regions of the human proteome.

**Figure 3.**
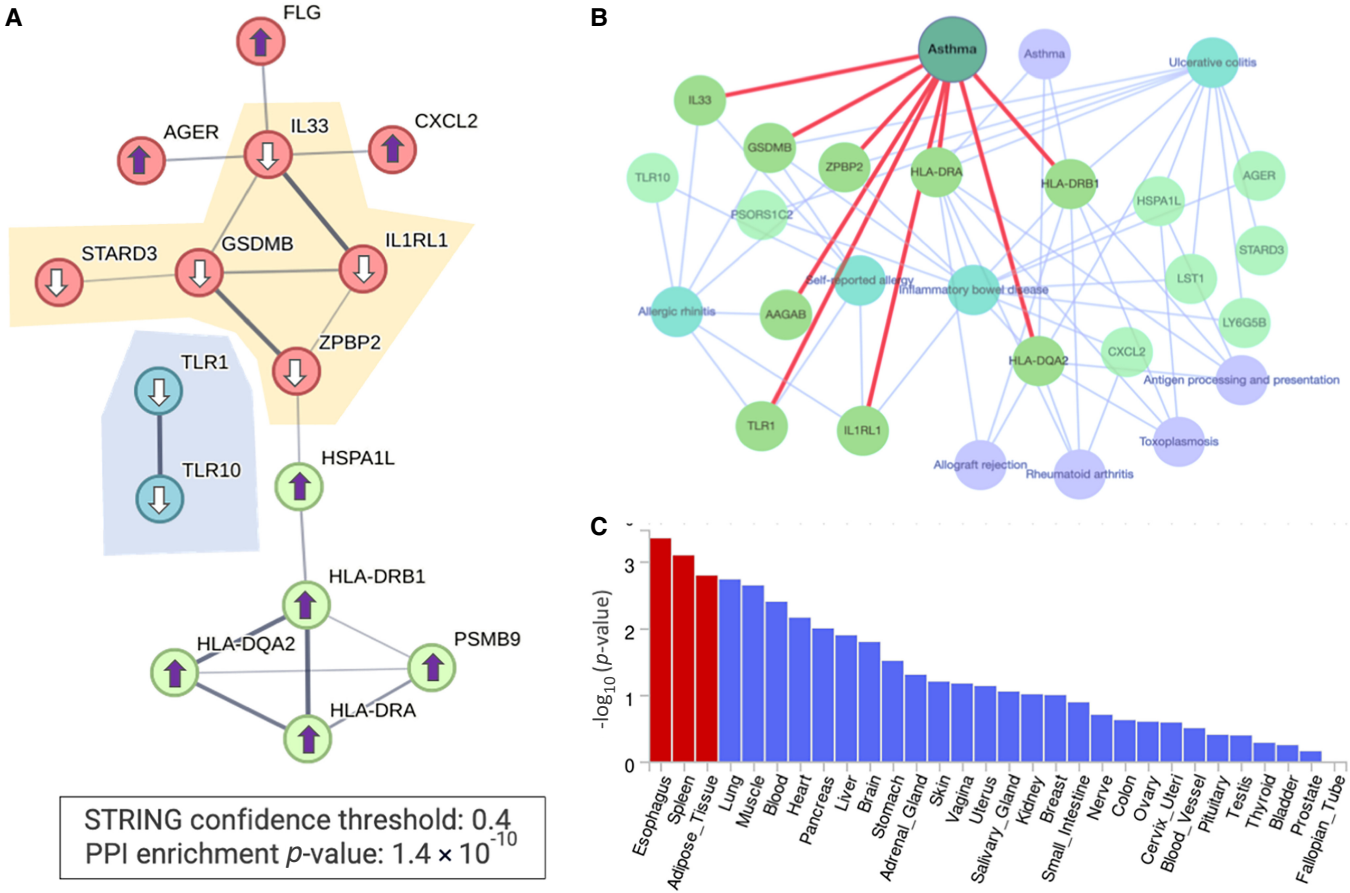
Functional enrichment of PWAS-associated genes of phenotype J45 (asthma). (*A*) STRING PPI network. The graph shows only significant connected genes (PPI confidence score >0.4, *P*-value for PPI network 1.4 × 10^−10^) along with their directionality (arrows up and down, depict increased and decreased risk, respectively). The subnetworks of genes that exert a decreased risk for asthma are highlighted. (*B*) A network view based on Enrichr-KB for the 27 PWAS-associated genes as input (Evangelista et al. 2023). The network consists of a knowledge graph based on the best match to the input gene list with the top findings from the GWAS catalog (light blue, underline) and from KEGG pathways (light purple). The red edges mark the associated genes identified by the GWAS catalog (2019) for asthma. (*C*) Statistical significance by tissue expression of the 27 identified PWAS-associated genes. The analysis combines all 27 differentially expressed genes (up- and downregulation) based on the GTEx database for 30 tissues as implemented in the FUMA platform (Watanabe et al. 2017). The significant tissues are colored red.

To answer this, we address the biological relevance of the 27 asthma-significant gene associations. Figure 3 displays an analysis of the results in Figure 2B. The highly significant protein–protein interaction (PPI) network (STRING confidence score >0.4; PPI *P*-value 1.4 × 10^−10^) (Szklarczyk et al. 2019) indicate direct connectivity for 15 of the 27 significant genes (22 according to a relaxed threshold of STRING confidence score >0.25). The most significant gene identified by PWAS for asthma is Gasdermin B (*GSDMB*). This gene is highly expressed in the lung bronchial epithelium in asthmatic patients. An alteration in the protein level of Gasdermin B indicates a reduced risk (Fig. 3A). In animal models, an increased level of *GSDMB* governs airway remodeling by increasing fibrosis in the absence of inflammation (Das et al. 2016).

The gene-based associations identified by PWAS Hub align with existing functional knowledge on asthma. Figure 3A also identifies *TLR1* and *TLR10*, which participate in the innate immune response that leads to cytokine secretion and an inflammatory response via MYD88 and NF-kB activation. Parallel molecular activation is exposed by IL33, which together with the IL1RL1 receptor triggers the NF-kB and MAPK pathways in target cells (Fig. 3A). *IL33* and *IL1RL1* were identified as PWAS-significant genes for asthma, in agreement with their cellular role in stimulating mast cells, basophils, eosinophils, and natural killer cells. The pathogenesis of eosinophilic asthma is further supported by the involvement of Filaggrin (FLG) in asthma severity in children and young adults (Palmer et al. 2007).

Figure 3B substantiates the high connectivity of the PWAS-associated genes by a knowledge graph of external resources. Several trends are evident: (1) Most genes are interconnected (20 of 27, a minimal request for ≥2 edges per gene). (2) The input genes highlight asthma and other inflammatory diseases from the GWAS catalog (e.g., allergic rhinitis, ulcerative colitis, and inflammatory bowel disease). (3) The immune cell dysregulation is corroborated by the top five KEGG pathways (Kanehisa et al. 2022) such as allograft rejection, antigen processing and presentation, and rheumatoid arthritis (RA). (4) Emphasizing HLA alleles in asthma development. The importance of the HLA profile was substantiated in asthmatic patients of various population origins (Mahdi et al. 2018). A statistical analysis of the knowledge graph derived from Enrichr-KB (Evangelista et al. 2023) is presented in Supplemental Table S1.

The gene-based associations identified by PWAS are independent of information derived from regulatory layers, such as tissue-specific expression. We assessed whether the asthma-associated gene list from PWAS is particularly linked to differential gene expression. Using GWAS FUMA (Watanabe et al. 2017), we examined the expression patterns of the 27 identified genes across 30 healthy tissues. Our analysis revealed that the esophagus, spleen, and adipose tissues are statistically significant compared to other tissues (Fig. 3C).

### Comparison of PWAS Hub results to the Open Targets benchmark

We compared the results of the PWAS Hub (hybrid mode, both) with the benchmark of OT (see Methods). Initially, we mapped the phenotypes with their ICD-10 code to the appropriate disease name in OT. Then, for each disease, we compared gene sets from OT to report the number of overlapping genes from the PWAS-significant gene list and OT top candidates. We created two OT overlapping genes: (1) Genes with a global OT score of >0.5 and (2) genes with genetic evidence and a GA score of >0.25.

**Figure 4.**
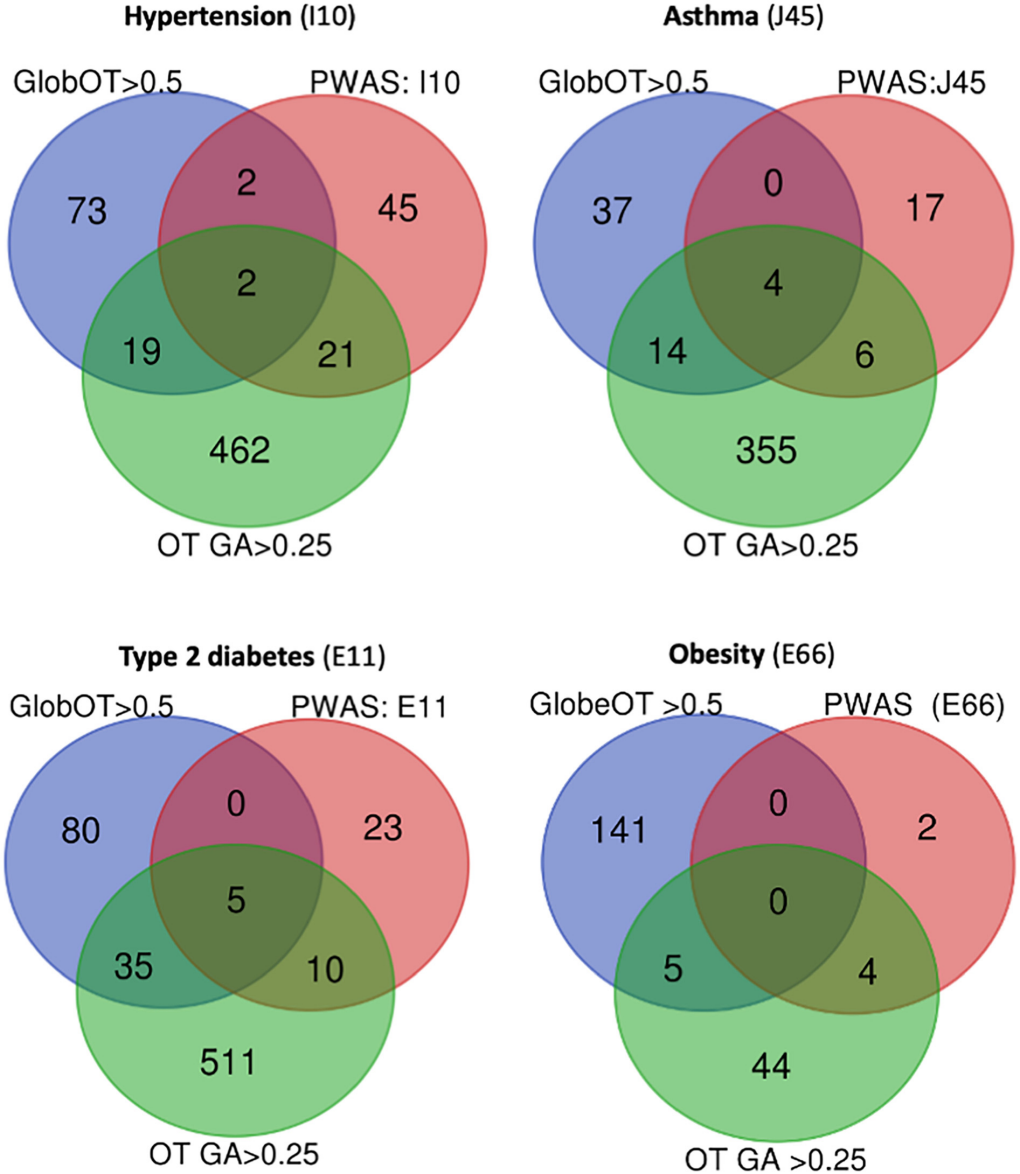
Comparative results of PWAS and Open Targets (OT) for multiple phenotypes. The Venn diagram indicates the number of overlapping genes for PWAS-significant genes, for OT by the global score >0.5, and for the results of GWAS as compiled by OT with GA score > 0.25. The results shown are for ICD-10 of hypertension (I10), asthma (J45), type 2 diabetes (E11), and obesity (E66). The statistical significance of the level of overlap determined based on the hypergeometric distribution test calculation.

We illustrate the findings for several of the phenotypes, including hypertension, asthma, type 2 diabetes, and obesity (Fig. 4). The OT platform reports 6738 associated genes for hypertension (ICD-10: I10), and 96 of them met the predetermined threshold (global OT >0.5). Only four genes (*AGT*, *DBH*, *NR3C2*, and *ADRA1D*) are shared between the 70 PWAS-significant genes and the 96 top OT gene sets. Hypergeometric distribution test indicated this overlap to be significant (Supplemental Table S2). When restricting the analysis to the top GA-scoring genes (total 1511; 504 genes with a GA score of >0.25), we observed a set of 23 overlapping genes (*P*-value 2.6 × 10^−3^). The overlap between the two sets from OT was quite modest (Fig. 4). While the number of significant genes by PWAS discovery is relatively small, the overlap between PWAS and GWAS gene lists remains significant for the majority of the phenotypes. Comparative analysis of PWAS and OT results (with Global and GA OT scores) is available in Supplemental Table S2.

### A gene-centric view can reveal a shared mode of action across diseases

**Figure 5.**
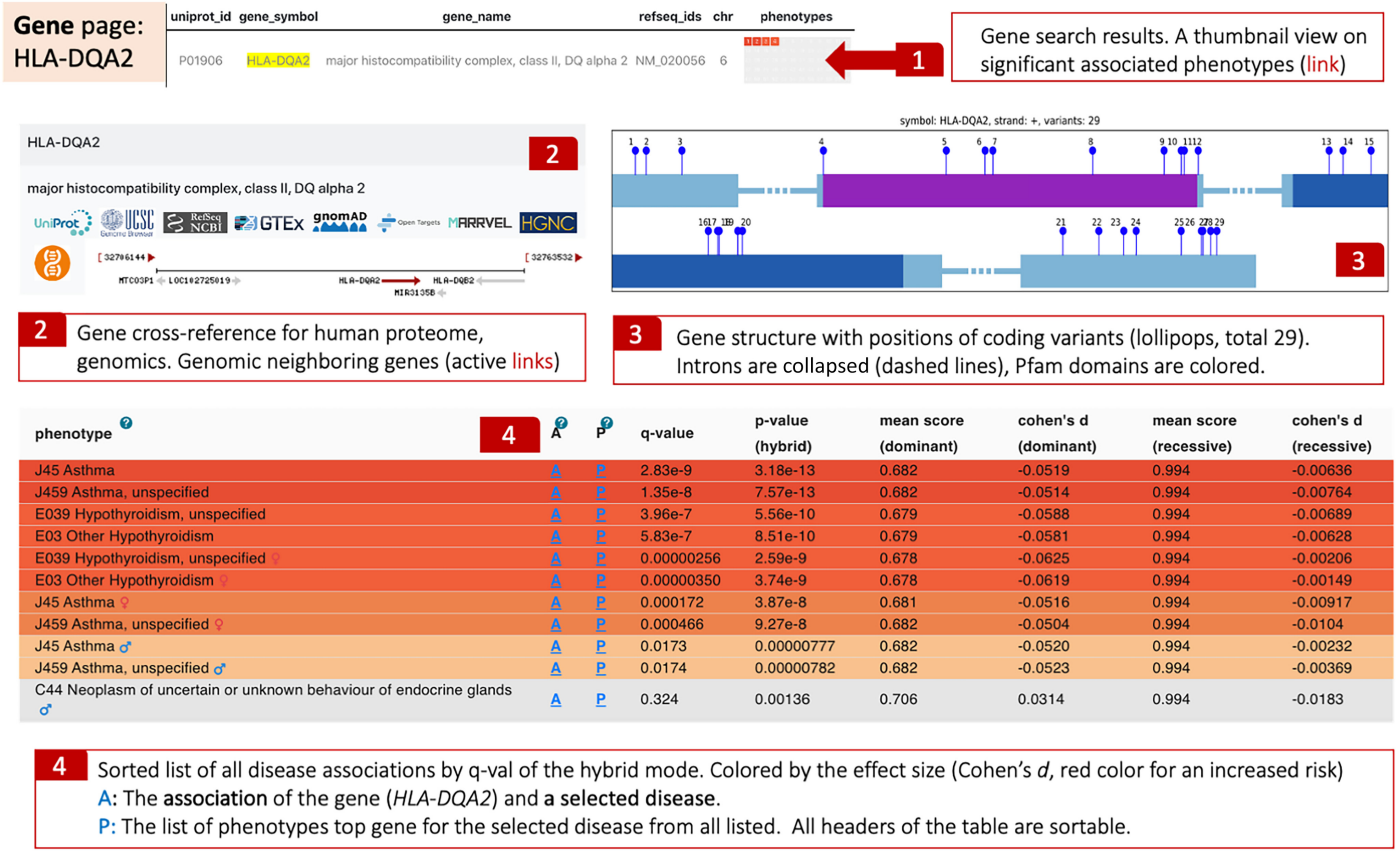
Gene-centric page. The search for a gene provides its protein and RefSeq IDs and a thumbnail-colored view of the associated phenotypes. The red color depicts the effect size and directionality of an increased risk (**1**). Cross-references to major resources include an active map of the genomic location of the gene (**2**). The numbered lollipops represent the coding variants that are included in the PWAS analysis, numbered along the protein length (**3**). The table summarizes the statistics of this gene with all other phenotypes and diseases. The colors depict the strength of the effect size and its directionality versus multiple phenotypes (**4**).

To further investigate the relevance of the PWAS Hub to gain insights on asthma etiology, we navigate to the gene view of *HLA-DQA2*. It is ranked second in the significant gene list and the most significant gene for an increased risk (Fig. 2B, *HLA-DQA2*, red color). Figure 5 shows the information presented on the “Gene page.” Note that one can navigate directly to gene-centric information using the search options by the gene name (free text), gene symbol (as in GeneCards), UniProt, RefSeq ID, or by its genomic location (Fig. 5, item 1).

The gene page (Fig. 5) provides a summary view of the statistical evidence for HLA-DQA2 and its association with any other listed diseases. We report a strong association that is restricted to asthma and hypothyroidism, two immune-related common diseases. Importantly, gene associations are indicative of increased risk in both diseases, consistent with having a shared genetic basis (Zucker et al. 2024). To further test the biological relevance of the subjected gene to asthma, the user can benefit from cross-reference resources. For example, the user can seek information on the gene association in the Open Targets (OT) genetic portal. List of genomic variations from exomes and genome sequencing within a healthy population (e.g., gnomAD [Koch 2020]). Cross-reference with GeneCards (Safran et al. 2021), UniProtKB provides a rich information on the gene, its protein product, and biological knowledge (UniProt Consortium 2019). We also included links to GTEx that highlight tissue specificity regulations and GO annotations (by AMIGO) for functional knowledge (Gene Ontology Consortium 2015).

**Figure 6.**
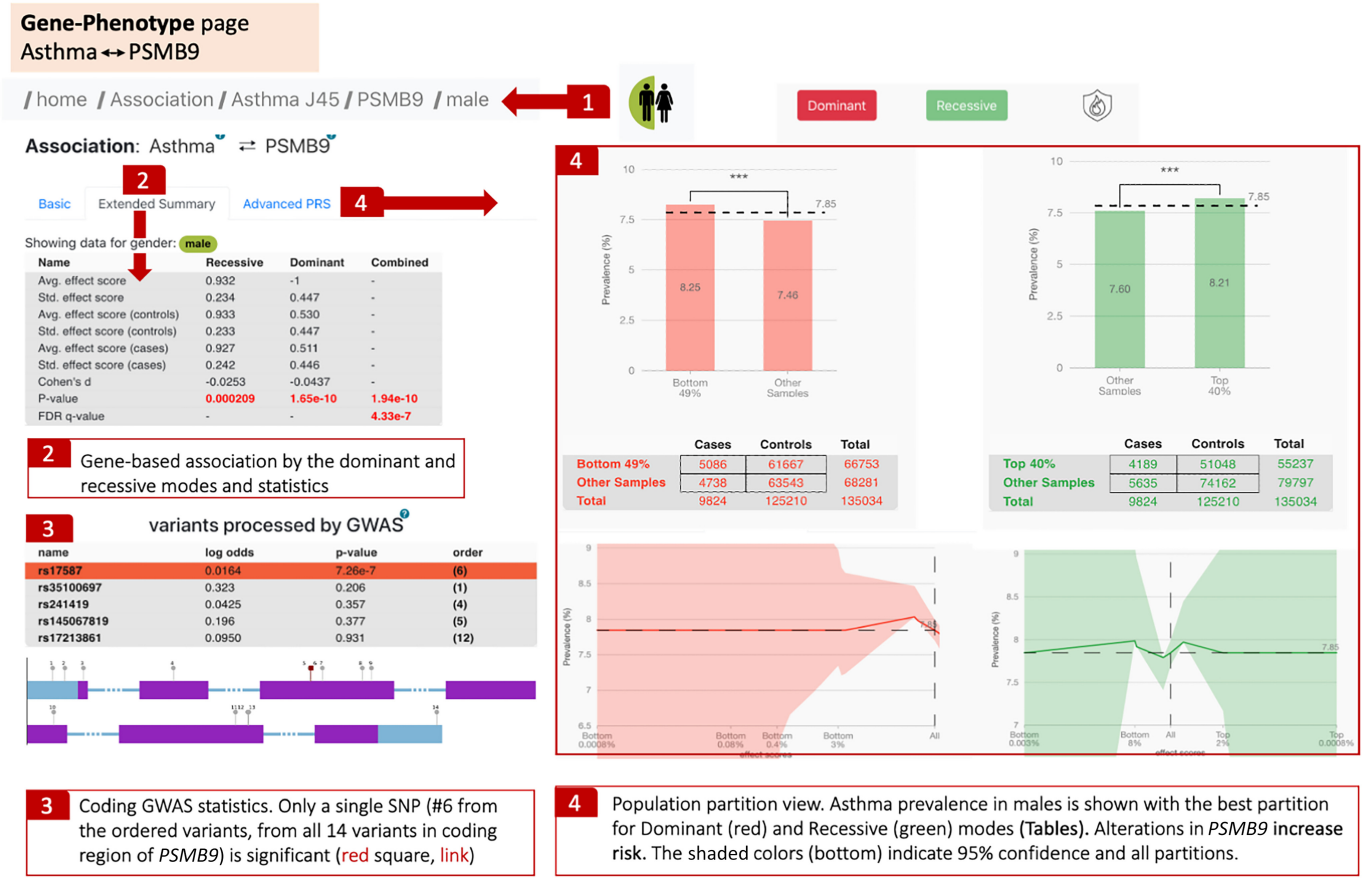
Gene–phenotype analysis page. The breadcrumb tool indicates the analysis is male-specific for asthma (**1**). Extended statistical information is shared in a table with a calculated *Q*-value for the hybrid heritability mode (**2**). The results of cGWAS and the list of significant variants along the sequence. Each variant is labeled by its sequential number along with the coding exons. Significant variant (#6) for asthma is marked by a red square (**3**). The population partition view of dominant and recessive gene-population analysis is shown with 2 × 2 contingency tables used to determine Fisher's exact statistics (**4**).

While we demonstrate that the user can start with a GOI or a DOI, the PWAS Hub application converges on the gene–phenotype analysis (Fig. 6). For asthma, when seeking the associated gene list by sex, only nine genes are listed as significant in males. We illustrate the results in asthma for the *PSMB9* gene (proteasome 20S subunit beta 9) that is ranked fourth in the male-dependent gene list. Figure 6 shows that the gene is highly significant for asthma in the hybrid mode (*Q*-value is 4.3 × 10^−7^).

An analytical view of the *PSMB9* gene in the context of asthma is shown via the population partition view by gene (Fig. 6). This view considers the partitioning of asthma-diagnosed individuals by the PWAS effect size of the gene. Specifically, we show that by considering the dominant mode of *PSMB9*, in 49% of the population group (i.e., males) that have the lower score for the effect size, the prevalence of asthma is slightly higher (Fig. 6, item 4, top-left panel). This is converted to an asthma prevalence of 8.25% (matched with 5086 male individuals with asthma), while the rest of the affected males have a lower prevalence of 7.46%. A similar trend is confirmed when considering the gene in recessive mode. This type of analysis refers to three main properties of the gene–disease associations: (1) what is the impact of modeling the gene by different heritability modes; (2) what percentage of the affected population can benefit from a simple population partition; and (3) what is the gap in prevalence when the population is partitioned below and above a cutoff partition line (i.e., for asthmatic males, the ratio is 1.106) (Fig. 6, item 4).

Yet, in addition to the gene-based view, PWAS Hub displays the underlying variants within the coding gene. In the case of *PSMB9*, while the coding region of the gene has 14 variants (Fig. 6, item 3), only a single SNP (rs17587, *P*-value 7.26 × 10^−7^) is statistically significant for the association with asthma. This SNP is very rare in the healthy population of gnomAD, with a minor allele frequency (MAF) of 1.4 × 10^−5^. This SNP increased the risk of RA for the ethnicity of Han Chinese in Yunnan (Yu et al. 2013) and vitiligo in the Saudi community (Mufti et al. 2021). These observations argue for shared mechanisms in asthma and other autoimmune diseases, as shown by analyzing the PWAS gene list (Fig. 3B).

### Gene–phenotype associations with sex groups

Finally, we present a global view that allows a direct comparison of the genetic effects on gene–phenotype associations by sex. To illustrate such a novel view, we compared the genes identified by females and males for asthma. Most of the signals in females and males are located in the MHC locus on Chr 6, which covers immunological risk factors for autoimmunity and many immune-related diseases.

**Figure 7.**
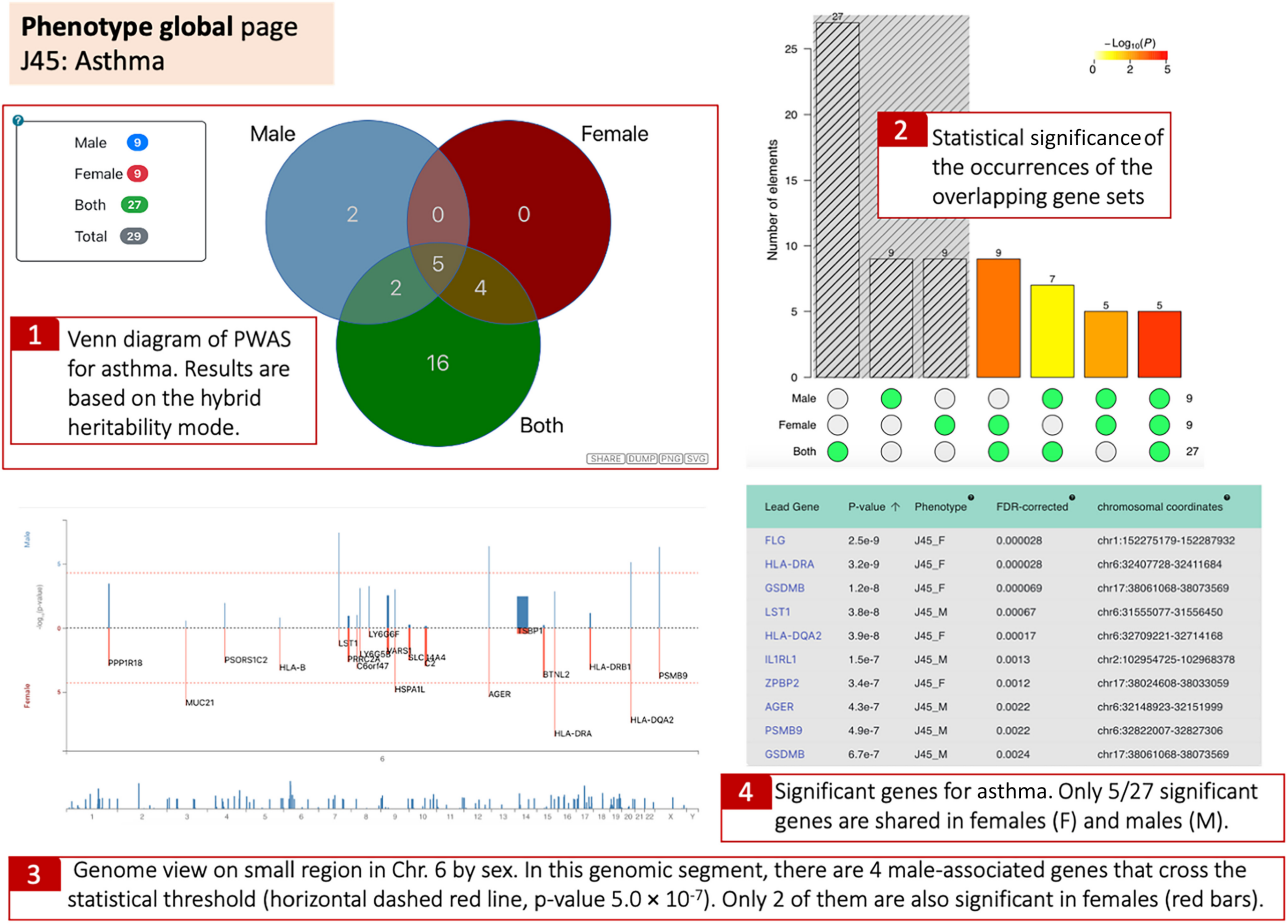
A collapsed view in the phenotype global page with a comparison of the genetic effects by sex. The Venn diagram shows the results for males, females, and both with the number of genes identified by PWAS Hub (**1**). A visual of a three-way Fisher's exact test is presented by the upset plot. The gray-shaded area is for the unions among the three subsets. The *P*-values are estimated to the *P*-value of having the exact subset of genes with respect to a random occurrence (**2**). Manhattan-like plot that maps the identified genes on the genome. The *y*-axis gives significance values for the enrichment for males (blue) and females (red). The *x*-axis depicts the chromosomal coordinates. A zoom-in option is implemented from a genomic overview. More information is available by popup options with hover over a gene name (**3**). A sortable summary table for each gene is shown for all genes according to their sex-stratified asthma (J45) (**4**).

Figure 7 (item 1) shows that the analysis by PWAS results in nine significant genes for males and nine for females. Only five of the genes are shared between the two sexes (see Venn diagram). It is also evident that there are no female-specific identified genes (Fig. 7, item 1), but two genes (*IL6R* and *MAP9*) were exclusively identified in males.

Notably, we have not set a threshold for sex-specific genes. Instead, we report all PWAS gene associations that meet the statistical criteria. Analysis of specific phenotypes revealed that for hypertension (I10), the gene effects in females are likely to differ from those in males. However, this was not observed for hypothyroidism, despite the different number of discoveries for each sex (Zucker et al. 2024). To support sex-specific hypotheses, further analysis, including permutation tests, would be required, which is beyond the scope of this study. We facilitate a comparison of gene overlap and uniqueness for each sex through inspection of the Venn diagram (Fig. 7).

Importantly, IL6 signaling through its receptor (IL6R) was associated with sex stratification as a risk factor for several autoimmune diseases (Hong et al. 2021). However, testing asthmatic and healthy young males failed to meet statistics with regard to IL6R (Rantala et al. 2011). More surprising is the finding that MAP9 expression is higher in males. The platform allows us to investigate the evidence supporting this finding. Specifically, there are three informative variants #19, #49, and #55 in males and none in females (out of the 57 along the coding region; http://pwas.huji.ac.il/gene-assoc/J45_M/MAP9). There are multiple observations that connect *MAP9* to cancer progression and hypertension, but reliable information in unavailable regarding *MAP9* polymorphism and asthma.

The PWAS Hub provides an entry point for interactively searching sex-specific results across the genome (Fig. 7, item 3). The genes are displayed by their genetic effects for males and females along the chromosomes using an interactive two-way Manhattan plot. We illustrate a snapshot of the genome browser for a small section of the MHC within Chr 6. There are four genes that are significant in males in this genomic segment, only two of them are also significant in females (*AGER* and *HLA-DQA2*; discussed in Fig. 6). In this region, *MUC21, HSPA1L*, and *HLA-DRA* are significant in female but not in male groups. The graphical results are presented along with a detailed tabular list of the sex-dependent informative genes.

## Discussion

PWAS detects gene–phenotype associations through the effect of variants on protein function, thus enhancing interpretability for complex diseases (Brandes et al. 2020). In PWAS, coding variants of each gene are aggregated and compiled into a unified gene effect score, under dominant and recessive heritability models. This is done for each individual. Specifically, in the last phase of PWAS, a straightforward statistical test is performed by comparing the mean and standard deviation of the partition to cases and controls per each of the heritable gene models. Therefore, irrespective of whether the PWAS score reflects genuine biology or not, it is guaranteed to minimize type I errors (false positive hits). Furthermore, as opposed to the variant additivity consideration routinely used in GWAS, the aggregating scheme in PWAS considers nonlinear interactions according to recessive or dominant models (see details in Brandes et al. [2020]). We found that in the case of cancer predisposition genes, the contribution of recessive inheritance is far greater than anticipated (Brandes et al. 2021).

It should be noted that PWAS Hub was applied on data from UKB that is prone to selection bias (Fry et al. 2017). Furthermore, the PWAS Hub has inherent limitations that could affect discovery. Most importantly, PWAS focuses only on the coding region of the gene, variants that affect regulation in noncoding regions will not be accounted for. Furthermore, the analysis used in the PWAS Hub was performed only on Caucasians from the UKB, which lowers the transferability of results to other population ancestries.

The PWAS Hub displays analysis for 99 phenotypes, each with an ICD-10 code, with at least 10,000 affected individuals of European origin. Accurate risk assessments are sensitive to sample size (Daetwyler et al. 2008). By selecting disease phenotypes with a minimal prevalence level of 3%–4%, we hope that biases due to cohort size are minimized. Some of the studied phenotypes include clinical conditions that are very broad (e.g., ICD-10 codes for K63.5, polyp of colon; Z51.1, chemotherapy; K59.0, constipation), many of which failed to identify significant genes and were excluded from the Summary page (Fig. 1). For most phenotypes, only a small number of PWAS-significantly associated genes were identified. Specifically, only 16 diseases have at least 10 significant genes (Summary page). For example, for Angina pectoris (ICD-10: I20), only three genes were identified as significant for males and none for females.

For most phenotypes, the number of PWAS-associated genes is quite small. We applied the QQ plot to nine representative phenotypes with >10 PWAS-significant genes each (Supplemental Fig. S1). The QQ plots show the deviation of observed *P*-values from the null hypothesis. Note that the majority of the observed values match the expected values (diagonal red line). The *P*-values that are more significant than expected are indicated by the points above the diagonal. We show that for most phenotypes, there is no evidence for inflation (λ values are close to 1.0) (Supplemental Fig. S1).

Many associated genes are reported for each phenotype using routine GWAS (as in the GWAS catalog or in the OT compilation). The ambiguity in assigning a variant to gene leads to a large number of false positives (Brandes et al. 2022). Based on the significant overlap (Fig. 4; Supplemental Table S2) between GWAS- and PWAS-based gene association results, the users are encouraged to apply both approaches as complementary methods. For example, in a recent work, we showed that the use of numerous association methods for hypothyroidism was beneficial to expose orthogonal aspects of the disease ontologies (Zucker et al. 2024).

We used asthma as a showcase due to its multiple etiologies and relatively strong heritability. In the PWAS Hub implementation for asthma, many of the genes were functionally interconnected (Fig. 3). The identified genes have a strong connection to psoriasis, type 1 diabetes, and hypothyroidism (Zucker et al. 2024). For some of the findings, biological evidence was previously reported. For example, TLR1 and TLR10 were identified as protective genes for asthma (Fig. 3A; Fore et al. 2020). Individuals with asthma have an increased expression of *TLR1* and *TLR10* in their airway epithelial cells compared to controls, suggesting these genes are asthma therapeutic targets (Wenger et al. 2023). Additionally, IL33 is positioned as a hub connecting several allergic pathologies (Fig. 3). While the PWAS discovery of *IL33* was entirely based on variants within the coding region, it was shown that a 5 kb region in the vicinity of the *IL33* locus is implicated in asthma. An enhancer-blocking element impacted chromatin conformation with a specific SNP (rs1888909) in this region that indirectly altered the *IL33* gene expression level (Aneas et al. 2021). Another finding concerns the RAGE protein (encoded by the *AREG* gene). Clinical data in humans suggest that *RAGE* is associated with asthma severity, especially in severe neutrophilic asthma (Perkins et al. 2021). We concluded that many of the PWAS-identified asthma-associated genes were implicated in the dysregulation of cell recognition in the innate immune system.

PWAS Hub has some drawbacks that limit its general utility. Most importantly, it is based on the coding regions that account for <5% of the variants collected. We plan to expand the utility of the PWAS Hub platform in the future by including disease classification according to PheWAS mapping (Denny et al. 2010). Under such a classification, we can increase the number of testable phenotypes. We also expect to include quantitative and categorized traits (e.g., measurement of blood tests, diastolic pressure) that signify higher discovery rate (Zucker et al. 2023). Another important additional piece of data concerns the variants located outside of coding genes and in regulatory regions. While interpretation is often missing, together with the assessment of rare variants from exome sequencing, can improve the discovery rate and propose attractive candidates for therapy. We anticipate to extend the utility of PWAS Hub in the future by incorporating rare and ultra-rare SNVs to the PWAS streamline. UKB provides a complete set of exome sequencing results that can be used in our streamline of gene-based association methods (Karczewski et al. 2022). With this notion, Genebass was developed as a webtool for assessing the contribution of rare coding variations across a wide range of traits (>4000) from the UKB (Karczewski et al. 2022). We expect that incorporating rare variants into the gene effect will enhance the discovery of PWAS without affecting gene-based statistical analyses. We propose that examining and navigating through the various pages and interconnected perspectives offered by the PWAS Hub can have substantial implications for personalized treatment strategies and improved disease management. Last, we illustrated that focusing on sex stratification is a valuable approach to highlighting sex-related disease etiology.

## Methods

### Data source

The input data for our study were sourced from the UKB, a large database that encompasses detailed medical, genotyping, and lifestyle information for ∼500,000 individuals aged 40–69 across the United Kingdom. The recruitment of participants took place between 2006 and 2010. To ensure consistency in our analysis, we focused on individuals of European origin (identified through data field codes 1, 1001, 1002, and 1003, and information on ethnic background via data field 21000). To account for genetic relatedness, we considered genetic ancestry (referred to as a genetic ethnic group, data field 22006) and randomly selected one representative from each kinship group, while removing other genetic relatives.

The disease classification in our study relies on the ICD-10 codes for the diagnosis. Similar to other association studies, PWAS uses case-control settings. The tested cohort included individuals that had been diagnosed by the main or secondary codes (UKB data fields 41202 and 41204, respectively), and the summary diagnosis code 41270. The latter covers the distinct diagnosis codes a participant has recorded across all their hospital inpatient visits, in either the primary or secondary position. The filtered cohort has ∼275,000 individuals of European origin. PWAS Hub covers diseases that are common and exceed 10,000 individuals in our filtered set, who have been diagnosed with a specific disease or condition (i.e., cases). Each cohort carrying a disease phenotype was divided into three sets: males, females, and both. In specific cases, only one of the sexes was analyzed. For example, the ICD-10 C50, malignant neoplasm of the breast, reported on 10,682 affected females, and only 77 affected males that were not analyzed.

### Variant inference by FIRM

The PWAS methodology assumes that variants in coding regions of a gene affect phenotypes by altering the biochemical function of the encoded protein. The FIRM (functional impact rating at the molecular level) is a pretrained ML model that estimates the extent of the damage caused to each protein for the entire proteome (Kelman et al. 2021). FIRM uses over 1100 numerical features to predict the effect score of any variant. For further details see GitHub (https://github.com/nadavbra/firm). The performance of FIRM reached an AUC of 90% (precision = 86%, recall = 85.5%) and an accuracy of 82.7% with respect to variants tagged as pathogenic/likely pathogenic in ClinVar (Landrum et al. 2020). Each nonsynonymous variant (e.g., missense, nonsense, frameshift, in-frame indel, and canonical splice-site variants) is assigned a functional effect score ranging from 0 to 1. Such scores capture the propensity of a variant to damage the gene's protein product. The predicted effect score of a variant is normalized so that zero reflects a complete loss of function (i.e., LoF mutation) and 1.0 represents a protein with no effect on protein function (i.e., synonymous). Note that the calculated effect score is agnostic to the phenotype, as the score reflects the impact on the function of the aggregated protein's biochemical damage.

PWAS reports its results on ∼18,050 coding genes. The current list of a complete human proteome is extracted from UniProtKB (Boutet et al. 2016) with over 20,000 proteins. About 2000 of them are not included in the analysis of PWAS. Among these unmapped genes are genes that are characterized by ambiguous mapping of UniProtKB to RefSeq. Examples are endogenous retrovirus groups (e.g., ERVK). The gene list that is not covered by PWAS is referred to in the FAQ section http://pwas.huji.ac.il/FAQ#noGeneSymbol. It is also available for direct download under http://pwas.huji.ac.il/api/file/uniprot.not_mapped.tsv.gz.

### Effect size of genes by heritability modes

Per-variant damage predictions are aggregated at the gene level for all listed variants. A protein-functional-effect-score is reported for dominant or recessive inheritance modes. The dominant inheritance model only requires a single allele to be affected, while for recessive inheritance, both alleles should be mutated for gene functionality to be affected. These scores are between 1 and 0, where a value of 1 represents no predicted loss of functionality and no effect on the phenotype for either dominant or recessive modes. PWAS also provides a combined model for the protein–phenotype association, which is termed the “hybrid” model. This model uses both the dominant and recessive effect scores of each participant as covariates in a logistic regression over the phenotype. To find gene associations, PWAS tests for a correlation between the functional alterations and the phenotype of interest using standard case-control statistical methods. To determine the size of the effect of a gene on PWAS, we applied a commonly used statistical measure of Cohen's *d* value. Cohen's *d* value (standardized mean difference, SMD) is the normalized difference in the mean gene effect score between cases and controls. It was calculated independently for either dominant or recessive effect scores. Further details on the ensemble of coding variants at a gene level are provided by considering the parameters for dominant and recessive scores and in Supplemental Methods (Brandes et al. 2020). In PWAS, positive values indicate a positive correlation with the gene effect scores, where higher effect scores represent less functional damage, thereby indicating the “protective” virtue of genes.

To account for potential biases (e.g., batch effect in data collection, residual population structure, age), we included a collection of 172 covariates with: sex (binary), year of birth (numeric), 40 principal components of the genetic data that capture the ancestry stratification were provided by the UKB (numeric), the UKB genotyping batch (one-hot-encoding, 105 categories), and the UKB assessment centers associated with each sample (binary, 25 categories). In this study, we included all 172 covariates, genotype batches, and assessment centers can be excluded to reduce the computational burden with minimal effect on discovery (Dor et al. 2023).

### Benchmark of Open Targets

The OT Platform scores target-disease associations and provides annotation information about targets, diseases, phenotypes, and drugs (Brown et al. 2018). The OT associations score aims to aggregate evidence relating to the link between a gene and a disease. The phenotypic representation of the disease is composed of many types of evidence, including animal models, literature, genetic evidence from GWAS (coined GA), clinical support from ClinVar, OrphaNet, drug, and expert view knowledge. For example, the Gene2Phenotype (G2P) is curated information based on consulting with clinical geneticists. The global OT association score combined direct and indirect evidence, and the available information was normalized to a single score (0–1). This score is used for ranking target-disease across different layers of evidence. We applied OT as a benchmark using the global OT score of >0.5 and the list of GAs compiled from a large collection of GWAS (Carvalho-Silva et al. 2019). We used the 24Q1 version (March 2024) that includes 63,226 targets, 25,817 diseases and phenotypes, and >7.8M target-disease associations.

### Coding GWAS analysis

While the PWAS effect scores use a gene-aggregation approach, we also performed a routine GWAS variant-independent approach under a similar case-control design, while restricting the variants to the coding region (coined coding GWAS, cGWAS). A total of 639,323 imputed variants (out of the 97,013,422 imputed variants) were included for analysis across the 18,053 protein-coding genes. Note that the set of variants used by PWAS and cGWAS is identical (Zucker et al. 2023). On average, there are 35.4 nonsense and missense mutations per coding gene.

The cGWAS associations were derived from PLINK 2.0 default logistic regression. The calculated *Z*-score specifies the effect size and directionality of the effect per variant. For GWAS, a positive *Z*-score indicates a risk variant due to the positive correlation between the disease and the number of alternative alleles. To minimize bias in comparing PWAS with cGWAS, we also included the same 172 covariates that were used in PWAS. In the PWAS Hub portal, we also report for each gene association the subset of significant variants from cGWAS that contributed to the association.

### A gene-centric risk by population partition

We tested the genetic signal of a disease in the entire population per gene. We examined the distribution of PWAS effect scores by considering all possible cutoffs (indicated by % of the cohort). For each gene–disease association study, we report the threshold that gave the best partition relative to the gene in the population presenting the disease in focus. For example, a threshold marked “bottom 10%” applies to effect-score values where the chosen threshold is 10% of the cohort with the lowest scores (i.e., the cohort decile with the greatest functional damage to the gene). Specifically, phenotype prevalence was calculated based on the 95% confidence interval using Wilson's score for binomial proportions (Kelman et al. 2021). This best-cut analysis is performed for females, males, and both sexes. Each gene–disease association was tested under recessive or dominant inheritance modes.

### Implementation of PWAS Hub and data integration

For a full discussion on the database schema and implementation, refer to the database page on PWAS Hub (http://pwas.huji.ac.il/Database). The schema with constrained tables for the major portal is shown under the menu bar “About.” To appreciate the quantitative scale, the number of rows for each main table is shown. Specifically, the main PWAS disease-statistical table includes 5.1M rows, the variant-disease table comprises 54.2M rows, and the gene list includes 18.1k. The gene variants table covers 225k informative rows.

### Ethical approval

The study was approved by the University Committee for the Use of Human Subjects in Research Approval number 12072022 (July 2022). This study uses the UK Biobank (UKB) application ID 26664 (Linial lab).

## Data access

All data generated and presented in the PWAS Hub platform are available in the data access guide link, http://pwas.huji.ac.il/API. This link is accessible from the “About” navigation bar under the API details item. The Python code for PWAS can be found at GitHub (https://github.com/nadavbra/pwas) and as Supplemental Code. PWAS is a free and open-source project available under the MIT License.

## Supplemental Material

Supplement 1

Supplement 2

Supplement 3

Supplement 4
