# Supplementary material for: PWAS Hub for exploring gene-based associations of common complex diseases
Source: Genome Res. 2024 Oct;34(10):1674–86. doi: 10.1101/gr.278916.123 (PMC11529988; doi:10.1101/gr.278916.123)
Supplement: Supplement 2 [file Supplemental_FigS1.pdf]

PWAS Hub for exploring gene-based associations of common complex diseases

Supplemental Figure S1

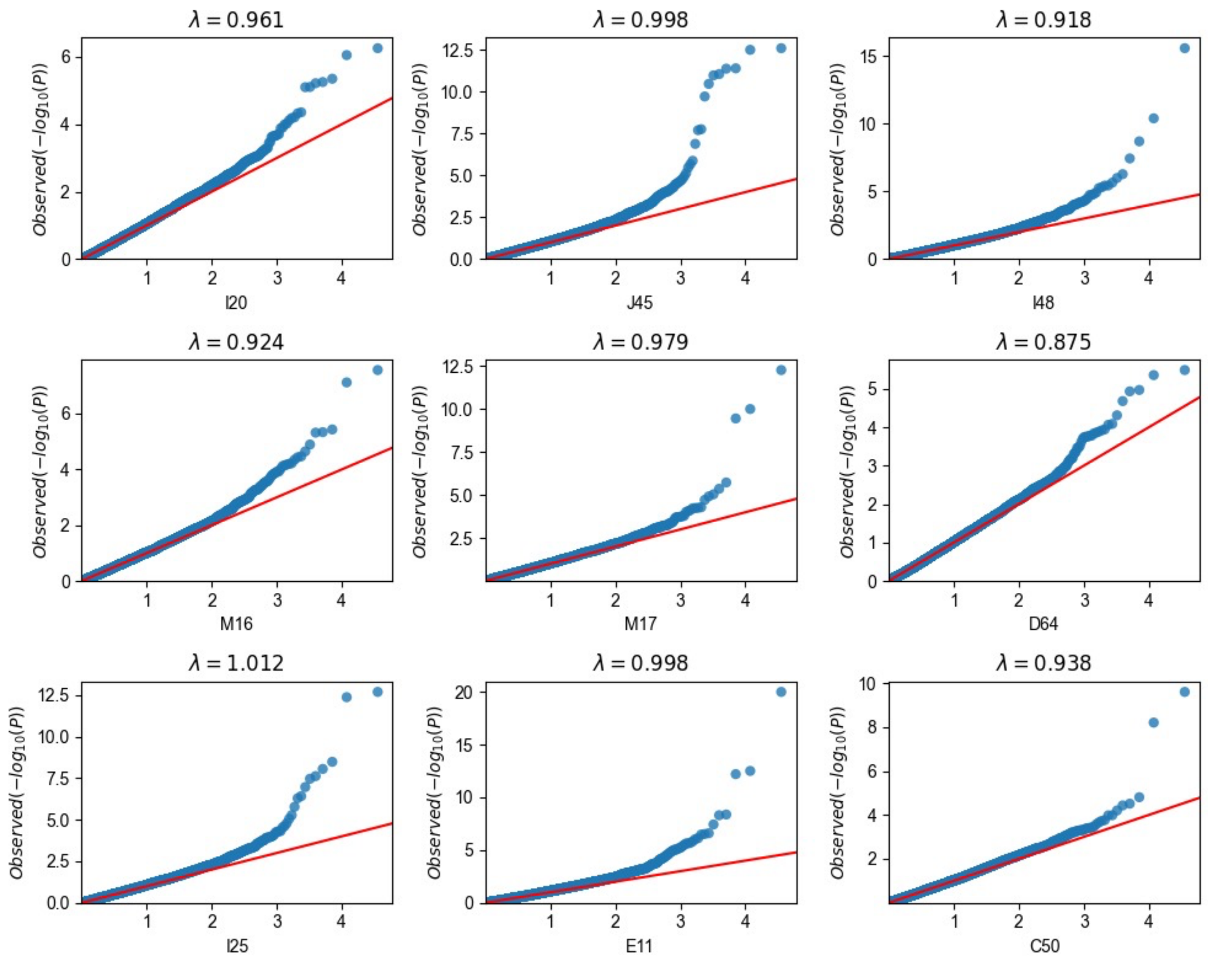

Figure S1. QQ plots of 9 representative phenotypes (indicated by the ICD-10). The phenotypes are selected by having >10 PWAS significant genes when analyzed for 'both'. The x-axis shows the -log10 of expected p-values of the association from the theoretical chi-square distribution, and the y-axis shows the -log10 of p-values from the observed chi-square distribution under FDR correction with α = 0.05. The genomic inflation factor λ is defined as the median of the observed chi-squared test statistic divided by the expected median of the corresponding chi-squared distribution. We show that most λ values are close to 1.0 (i.e., no evidence of inflation), exception is QQ plot for D64 (other anemia) with λ of 0.875.
